# Optimization of Properties of Calcium Hexaluminate-Based Insulating Castables with Calcium Aluminate Cement

**DOI:** 10.3390/ma18102354

**Published:** 2025-05-19

**Authors:** Yufeng Xia, Cuijiao Ding, Wei Luo, Haizhen Yang, Wenjie Yuan

**Affiliations:** 1 State Key Laboratory of Advanced Refractories, Wuhan University of Science and Technology, Wuhan 430081, China; xiayufeng0515@163.com (Y.X.); yhz9801@163.com (H.Y.); 2 Energy and Environment Research Institute, Baosteel Central Research Institute, Wuhan 430080, China; e11079@baosteel.com (C.D.); e80605@baosteel.com (W.L.); 3 National-Provincial Joint Engineering Research Center of High Temperature Materials and Lining Technology, Wuhan University of Science and Technology, Wuhan 430081, China; 4 Joint International Research Laboratory of Refractories and Metallurgy, Wuhan University of Science and Technology, Wuhan 430081, China

**Keywords:** CA_6_-based insulating castables, calcium aluminate cement, properties, fractal dimensions of pores

## Abstract

In the context of global energy scarcity, thermal insulation castables have garnered significant attention from the steel industry to reduce energy consumption. To optimize the performance of calcium hexaaluminate (CA_6_)-based insulating castables, a systematic comparative study was conducted on the influence of varying amounts of calcium aluminate cement (CAC) incorporated into the castables. The results indicated that the addition of more CAC could increase the initial flowability of the castables with an air-entraining agent (AEA). Conversely, the flowability of the castables containing alumina bubbles continuously decreased after 30 min and 60 min. The apparent porosity of castables with only added AEA and alumina bubbles after being dried at 110 °C and treated at 1300 °C presented a decreasing trend as CAC content increased. Under the joint action of AEA and alumina bubbles, the amplification in porosity of castables treated at 1300 °C was positively correlated with the amount of CAC. The increase in CAC content could enhance the strength of samples, with a particularly notable improvement observed in castables prepared with the addition of AEA. For castables prepared with AEA and CAC contents of 9 wt.%, the cold modulus of rupture and cold crushing strength after heat treatment at 1300 °C were 17.5 MPa and 80.5 MPa, respectively. The thermal conductivity of castables presented non-monotonic change with the increase in CAC content. The effect of elevated CAC content on the pore fractal dimension of castables depended on the pore-forming methods. Grey correlation analysis (GCA) demonstrated that pore sizes in the range of 500–1000 nm, pore fractal dimensions, and pore sizes less than 500 nm had the highest degrees of correlation with CMOR, CCS, and thermal conductivity, respectively.

## 1. Introduction

The water-cooled beam and post of the reheating furnace are the load-bearing component for steel slabs entering and exiting the furnace and achieving heating inside the furnace. The outermost castables of water-cooled beams and posts may crack and peel off under harsh usage conditions. Looking back at the development of refractories for the reheating furnace of Nippon Steel, the support lining of the beam and post evolved from bricks before the 1970s to plastic mixes in the 1970s, ceramic fibers and insulation castables in the 1980s and 1990s, and the current calcium hexaaluminate castables and prefabricated blocks [1]. Enhancing the thermal insulation properties of refractories utilized in beams and posts is essential for minimizing heat loss in reheating furnaces attributed to cooling water. The challenge for this kind of material lies in balancing thermal conductivity and strength.

As the compound with the highest Al_2_O_3_ content in the CaO–Al_2_O_3_ binary system, CA_6_ (CaAl_12_O_19_) has wide application prospects in high-temperature industry due to its low solubility to molten metal and slag, low wettability, exceptional stability under reduced atmosphere and excellent alkaline corrosion resistance [2,3,4]. Platelet CA_6_ grains show anisotropic growth, which can improve the toughness and thermal shock resistance of castables [5,6]. Porous CA_6_ has a low thermal conductivity (0.4–0.6 (W/m∙K)) [7]. The thermal expansion coefficient of CA_6_ is 8.0 × 10^−6^/°C, which is close to that of Al_2_O_3_ at 8.6 × 10^−6^/°C, so that there is no expansion mismatch between CA_6_ and Al_2_O_3_ with any ratio [8]. The incorporation of CA_6_ as an aggregate into corundum spinel castables can give full play to the toughening effect while controlling the volume stability [9,10]. Substituting CA_6_ particles for tabular corundum as aggregates enhanced strength at room and high temperatures, provided thermal shock resistance, and reduced linear dimensional changes post-firing at 1550 °C [11]. Notably, this enhancement became more pronounced with a decrease in CA_6_ particle size. The most pores in CA_6_ particles with higher porosity were micropores, which can strengthen the bond between aggregate and matrix, improving the strength of samples. Xu [12] developed CA_6_ castables composed of CA_6_ (aggregate, fine powder) and Al_2_O_3_ powders as the primary raw materials and calcium aluminate cement as the binder for the permanent layer of a ladle. The thermal insulation effect of CA_6_ castables was better due to their lower thermal conductivity of CA_6_ castables (0.631 W/(m∙K) at 500–1000 °C) compared with high-alumina castables (1.028 W/(m∙K) at 1000 °C).

Calcium aluminate cement (CAC) is one of the raw materials that has been widely used as a binder in monolithic refractories [9,13,14,15]. CAC-bound castables are widely used in high-temperature industrial fields, particularly iron and steel metallurgy, due to their high early strength, excellent erosion resistance, and wear durability [16,17,18]. During the casting, molding, hardening, demolding, drying, and subsequent heating and firing processes, a series of changes occur in the phase and structure of the hydration products, thus affecting the macroscopic mechanical properties of CAC-bonded castables. Firstly, a hydration reaction of CAC-bonded castables occurs during the construction process, forming hydration products CAH_10_, C_2_AH_8_, and so forth, which provide the castables with sufficient strength after demolding. Subsequently, during the drying phase, free water is volatilized. The dehydration and decomposition of the hydration products during the heat treatment process at elevated temperatures results in an increase in porosity, a loosening of the structure, and a reduction in strength. Finally, a heat-treating reaction occurs inside the matrix of the castables at high temperatures [19,20,21,22].

The impact of various pore-forming methods on the properties of CA_6_ castables was investigated. It was demonstrated that the addition of an air-entraining agent was more effective in generating pores within the castables, while the incorporation of alumina bubbles could significantly enhance the strength of the castables. Based on these findings, CA_6_ castables with low thermal conductivity and sufficient strength have been developed [23]. For cement-bonded castables, cement content control is a key issue. The formation of excess CA_6_ could result in increases in porosity, and consequently, mechanical strength deterioration and a decrease in penetration resistance. For alumina–magnesia castables, a better balance among properties including expansion, creep resistance, and thermal shock resistance can be achieved with an intermediate CAC content (4 and 6 wt.%) [13]. The selection of a suitable CAC content is significant for the performance and working life of CA_6_-based insulating castables. The purpose of this study was to determine the definitive amount of CAC so as to optimize the performance of CA_6_-based insulating castables with low thermal conductivity and sufficient strength. In this work, the effects of alterations in CAC content on the physical properties, thermal conductivity, and pore size of CA_6_-based insulating castables prepared through different pore-forming methods were investigated. The results provide a reference for the optimization of properties for CA_6_-based insulating castables.

## 2. Materials and Methods

### 2.1. Raw Materials

The raw materials used in the experiment were CA_6_ (5–3 mm, 3–1 mm, 1–0 mm, ≤0.074 mm, RECAG-90LD, Shengchuan, Zibo, China), alumina powder (AMA-40, AMA-10, Smile, Hongan, China), CAC (Secar71, Imerys, Tianjin, China), alumina bubbles (0.2–0.5 mm, Sanmenxia Chaochang, Sanmenxia, China), water reducing agent (WSM-M, Smile, China), disodium dodecyl succinate as air-entraining agent (AEA2066, Dongguan Xianchuang, Dongguan, China), and hydroxyethyl methyl cellulose as foam stabilizer (WALOCEL™ MT 400 PFV, Dow Chemical Company, Midland, MI, USA).

### 2.2. Preparation Method

The samples were prepared according to the unshaped refractory sample preparation method (YB/T 5202.1-2003) [24]; the formulations of the samples are presented in Table 1. The dry mixture was initially blended in a cement mortar mixer for 2 min. Subsequently, deionized water was gradually introduced within 1 min, with continuous stirring of the total mixture for 2 min. The stirred mixture was poured into molds, one with 25 mm × 25 mm × 150 mm and another with ϕ180 mm × 20 mm, forming by flowability. The samples were cured at 25 °C and 80% humidity for 24 h and then dried at 110 °C for 24 h. The dried samples were heat-treated at 1000 °C and 1300 °C, for 3 h at each temperature.

### 2.3. Characterization and Testing

The phase composition of the samples was characterized using an X-ray diffractometer (XRD, X’pert Pro MPD, Philips, Almelo, The Netherlands). Using Ni as filter and Cu–K_α_ as an X-ray source, the wavelength was 0.15406 nm, the scanning range spanned from 10 to 90° (2θ), the step length was 0.03°/s, the test tube voltage of the instrument was 40 kV, and the tube current was 40 mA. The flowability of the castables was measured according to GB/T 4513.4-2017 standard [25]. The flowability at 0 min, 30 min, and 60 min was tested as T0, T30, and T60 respectively. The bar samples, subjected to heat treatment at various temperatures, were evaluated for their cold crushing strength (CCS) according to the GB/T 5072-2008 standard [26]. Additionally, the cold modulus of rupture (CMOR) was tested using the three-point bending method based on GB/T 3001-2017 standard [27]. According to GB/T 2997-2015 standard [28], the apparent porosity and bulk density of the samples were tested based on the principle of the Archimedean method with water as the medium. Permanent linear change in the sample after heat treatment was measured according to GB/T 5988-2022 standard [29]. The pore size distribution of samples treated at 1300 °C was characterized by mercury intrusion porosimetry (MIP, AUTOPORE 9500, Micromeritics’ instruments, Atlanta, GA, USA). According to YB/T 4130-2005 standard [30], the thermal conductivity of the samples was measured with a flat plate thermal conductivity tester (PBDR-03P model, Precondar, Luoyang, China).

The fractal dimension *D* is an indicator of surface roughness using fractal geometry concepts. The pore structure can be understood as a fractal entity with regard to pore surface area. The surface fractal dimensions of porous media can be obtained by mercury intrusion data. Mercury was injected into large-sized pores, and as pressure increased, mercury continuously occupied secondary small pores. The equation is as follows [31]:

(1) $$D=4+\ln\left(\frac{dV}{dp}\right)/lnp$$

*D* is the fractal dimensions; *V* is the pore volume measured by MIP (mL/g); *p* is the pressure (MPa) applied by MIP.

## 3. Results

### 3.1. Phase Composition

**Figure 1:**
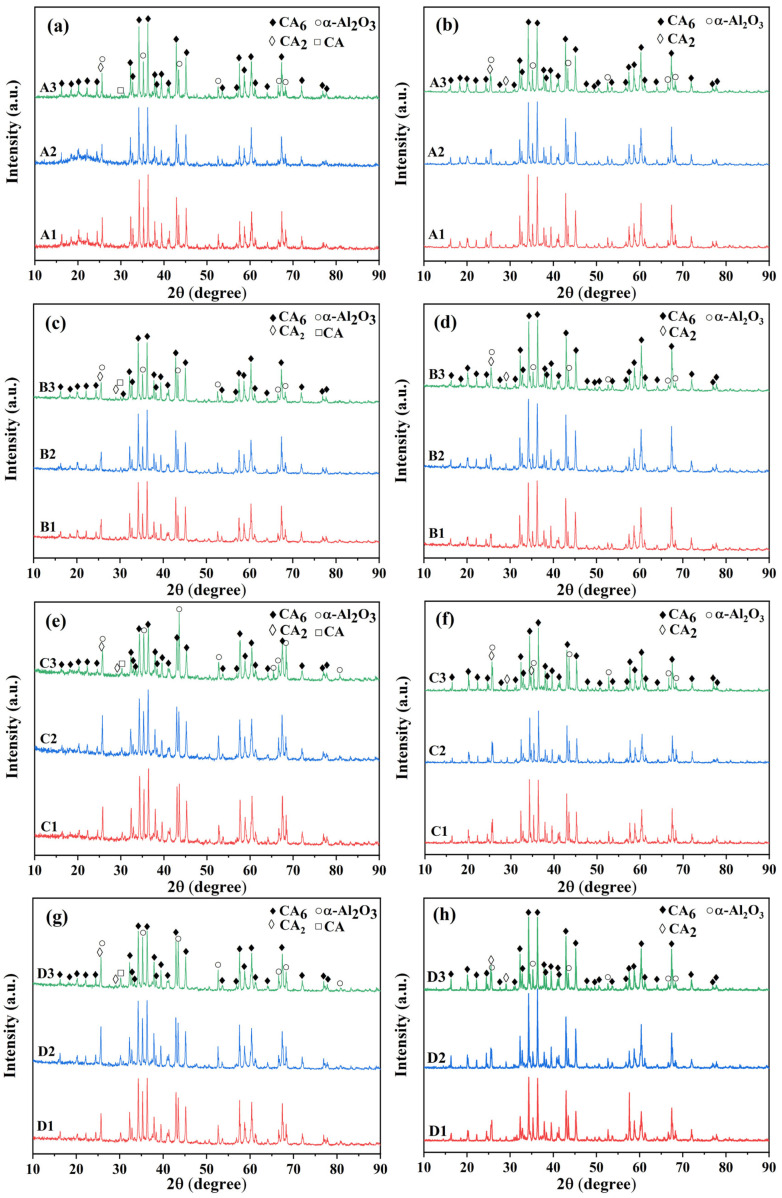
XRD patterns of CA_6_-based insulating castables after heat treatment at 1000 °C (**a**,**c**,**e**,**g**) and 1300 °C (**b**,**d**,**f**,**h**).

Figure 1 presents the XRD patterns of castables mixed with different CAC contents. It can be seen from the spectra that the main phases of castables after 1000 °C are CA_6_ and α-Al_2_O_3_, while the contents of the CaAl_4_O_7_ (CA_2_) and CaAl_2_O_4_ (CA) phases are very low. After treatment at 1300 °C, the main phases of castables continued to be CA_6_ and α-Al_2_O_3_; the CA phase disappeared, due to the relatively low heat treatment temperature, while there was still a residual CA_2_ phase. After treatment at temperatures of 1000 °C and 1300 °C, because of the unchanged content of alumina micro powder, the relative intensity of the α-Al_2_O_3_ diffraction peak decreased as the cement content increased. The mineral phase in CAC reacts with alumina to form CA_6_ phase in situ, so the change in CAC content had little effect on the overall phase composition of the castables.

### 3.2. Flowability

**Figure 2:**
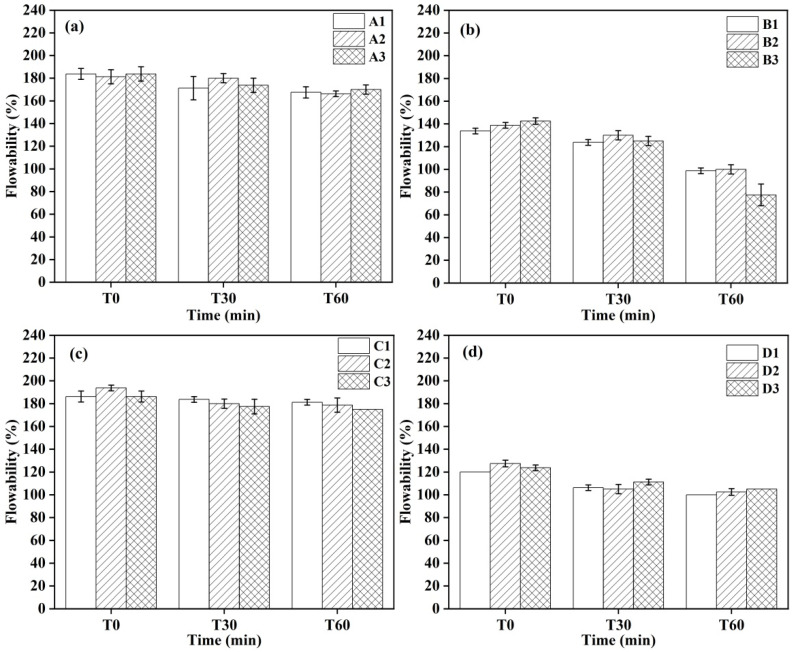
Flowability of CA_6_-based insulating castables: (**a**) A1–A3, (**b**) B1–B3, (**c**) C1–C3 and (**d**) D1–D3.

Figure 2 illustrates the flowability of castables formulated with different CAC contents. The results indicated that the incorporation of CAC enhanced the flowability of castables devoid of additives, with an optimal flowability achieved with a CAC content of 10 wt.%. Furthermore, the increase in CAC content also increased the initial flow value of castables with an air-entraining agent added. However, it also accelerated the rate of decline in flowability. A detailed analysis of the flow values at T60 revealed that sample B3, containing 10 wt.% CAC, exhibited the lowest flow value of 77.5%. Conversely, sample D1, formulated with 8 wt.% CAC, maintained the highest flow value at T60 of 100%. Notably, sample C2, with 9 wt.% CAC, presented the highest initial flow value of 193.75% at T0. The variation in CAC content had a minor impact on castables prepared by simultaneously adding an air-entraining agent and alumina bubbles.

### 3.3. Bulk Density and Apparent Porosity

**Figure 3:**
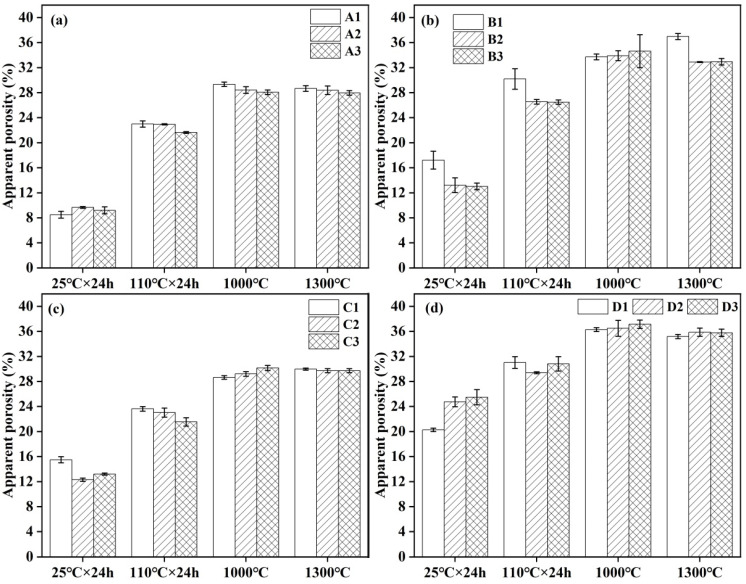
Apparent porosity of CA_6_-based insulating castables: (**a**) A1–A3, (**b**) B1–B3, (**c**) C1–C3 and (**d**) D1–D3.

**Figure 4:**
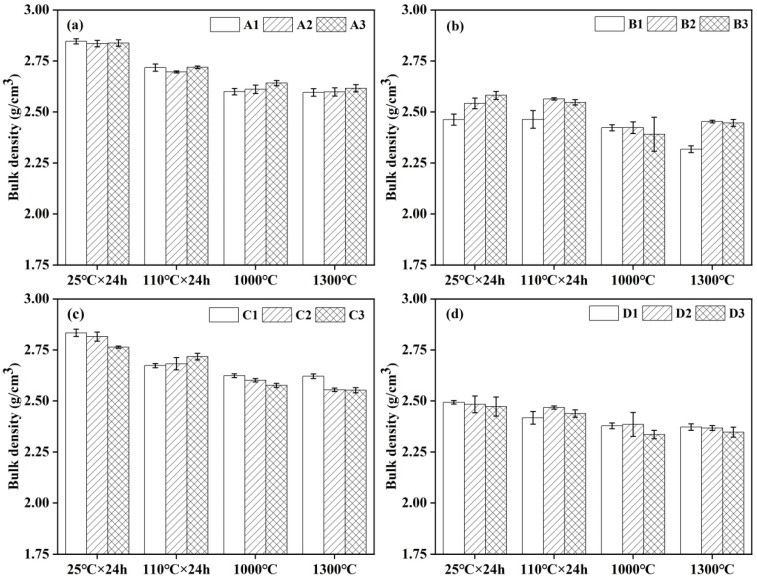
Bulk density of CA_6_-based insulating castables: (**a**) A1–A3, (**b**) B1–B3, (**c**) C1–C3 and (**d**) D1–D3.

The influence of the cement content on the apparent porosity and bulk density of the castables is illustrated in Figure 3 and Figure 4. As shown in Figure 3a, an increase in cement content resulted in a decrease in the apparent porosity of samples prepared without additives dried at 110 °C as well as after treatment at 1000 °C and 1300 °C. The apparent porosity of the samples increased slightly, with minimal impact on the bulk density (Figure 4a). From Figure 3b,c, the apparent porosity of the castables prepared with an air-entraining agent and alumina bubbles separately was reduced with the increase in cement content, except for samples treated at 1000 °C. This was attributed to more fine pores formed after hydration and dehydration of cement and sintering at 1300 °C. The bulk density of samples with alumina bubbles (Figure 4c) had little change, with sample C1 containing 8 wt.% cement achieving the maximum bulk density of 2.62 g/cm^3^ after treatment at 1300 °C. The magnitude of the change in apparent porosity of samples D2 and D3 enlarged after demolding, as seen in Figure 3d. Following heat treatment at 1000 °C, the apparent porosity of all samples increased. After heat treatment at 1300 °C, the apparent porosity of all samples decreased, with D1 showing the largest reduction. The variation in the apparent porosity and bulk density of castables was most pronounced after demolding at 25 °C and drying at 110 °C due to the cement hydration process playing a dominant role during these two stages. However, reaction and sintering became the dominant factors when samples were treated at 1000 °C and 1300 °C.

### 3.4. Microstructure

**Figure 5:**
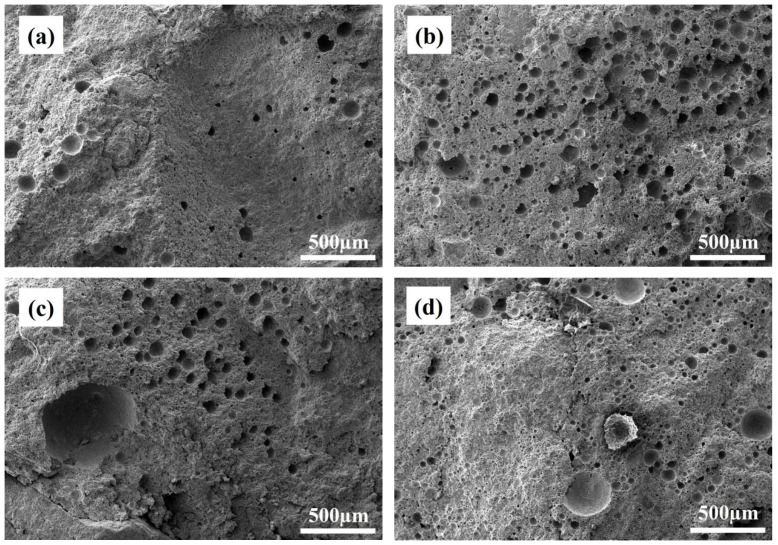
SEM images of CA_6_-based insulating castables with 10 wt.% CAC after treatment at 1300 °C: (**a**) A3, (**b**) B3, (**c**) C3, (**d**) D3.

Figure 5 shows the microstructure of castables with 10 wt.% CAC. The apparent porosity of sample A3 was relatively low, as seen in Figure 5a. The formation of spherical pores was mainly related to excess water, which would evaporate at high temperatures. From Figure 5b, it can be seen that pores were more widely distributed in sample B3, which was a consequence of the addition of an air-entraining agent. Larger pores (greater than 400 µm) were left after the spalling of alumina bubbles in sample C3, as shown in Figure 5c. As a whole, there were fine pores derived from the air-entraining agent and separate pores with a size larger than 100 µm from the addition of alumina bubbles in sample D3 (Figure 5d). In broken alumina bubbles, block crystals of alumina different from CA_6_ can be observed.

### 3.5. Strength

**Figure 6:**
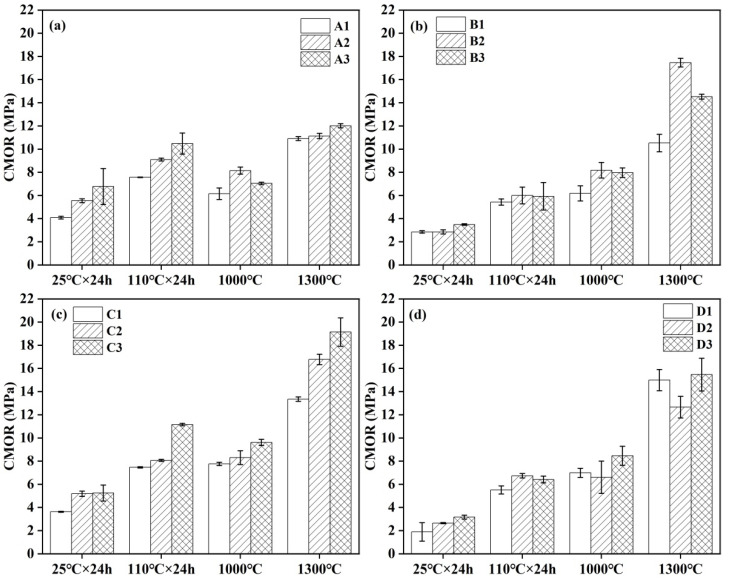
CMOR of different groups of CA_6_-based insulating castables: (**a**) A1–A3, (**b**) B1–B3, (**c**) C1–C3 and (**d**) D1–D3.

**Figure 7:**
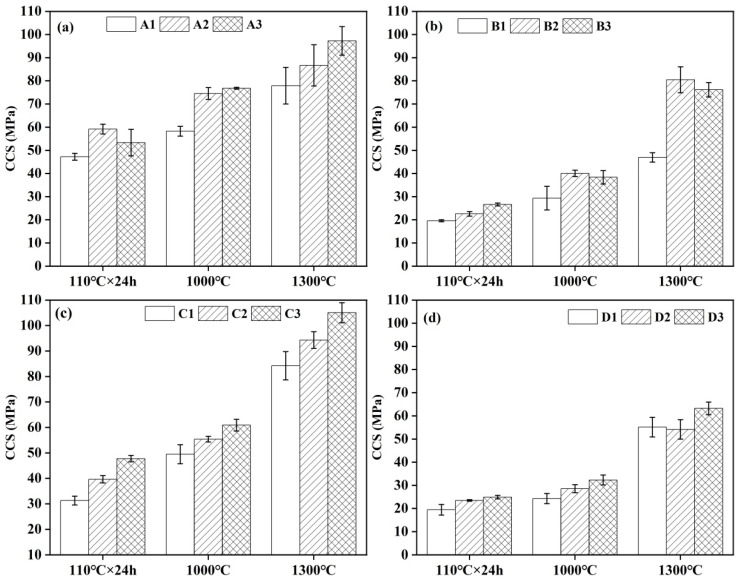
CCS of different groups of CA_6_-based insulating castables: (**a**) A1–A3, (**b**) B1–B3, (**c**) C1–C3 and (**d**) D1–D3.

The CMOR and CCS of different castables are presented in Figure 6 and Figure 7, respectively. In general, the strength of most samples was augmented with increments in the CAC content. This enhancement in strength, particularly for samples after demolding and drying at 110 °C, may be attributed to the hydration process of the cement. Elevated cement content led to an increased formation of hydration products, which are calcium-aluminum-hydrate (C-A-H). The continuous growth of these hydration products facilitated the formation of additional three-dimensional network structures among particles, thereby enhancing the early-age strength of the castables. A comparative analysis of samples revealed that the CMOR and CCS of the samples exhibited varying degrees of improvement with increasing CAC content (Figure 6a and Figure 7a). Notably, after treatment at 1300 °C, sample A3, containing 10 wt.% CAC, presented the highest CMOR and CCS, reaching 12.0 MPa and 97.3 MPa, respectively. It was noteworthy that the CMOR of samples A1–A3, treated at 1000 °C, was lower than that of the samples dried at 110 °C. When samples were heated at 1000 °C as a medium-temperature stage, the cement hydration products in the castables underwent dehydration and decomposition reactions during the heating process, leading to a reduction in strength. Conversely, upon heat treatment at 1300 °C, a pronounced sintering effect was observed in the samples, resulting in a marked improvement in their CMOR and CCS.

In the castables prepared with the addition of air-entraining agent (AEA), after heat treatment at 1000 °C and 1300 °C, both the CMOR and CCS exhibited a trend of initial increase, followed by a decrease (Figure 6b and Figure 7b). Specifically, sample B2, with a cement content of 9 wt.% and heat treatment at 1300 °C, exhibited the highest CMOR and CCS values, which were 17.5 MPa and 80.5 MPa, respectively. However, the CCS value of castables fired at 1400 °C specified in the national standard GB/T 22590-2021 is only 55 MPa. In castables prepared with the addition of alumina bubbles, after heat treatment at 1000 °C and 1300 °C, the strength of the samples increased with the increase in cement content (Figure 6c and Figure 7c).

For the castables prepared with the simultaneous addition of air-entraining agents and alumina bubbles, the CMOR of samples after heat treatment at 1000 °C and 1300 °C showed a trend of initial decrease, followed by an increase (Figure 6d). Specifically, sample D3, with a cement content of 10 wt.% and heat treatment at 1300 °C, had the highest CMOR and CCS values, which were 15.47 MPa and 63.23 MPa, respectively (Figure 6d and Figure 7d).

### 3.6. Pore Size Distribution

**Figure 8:**
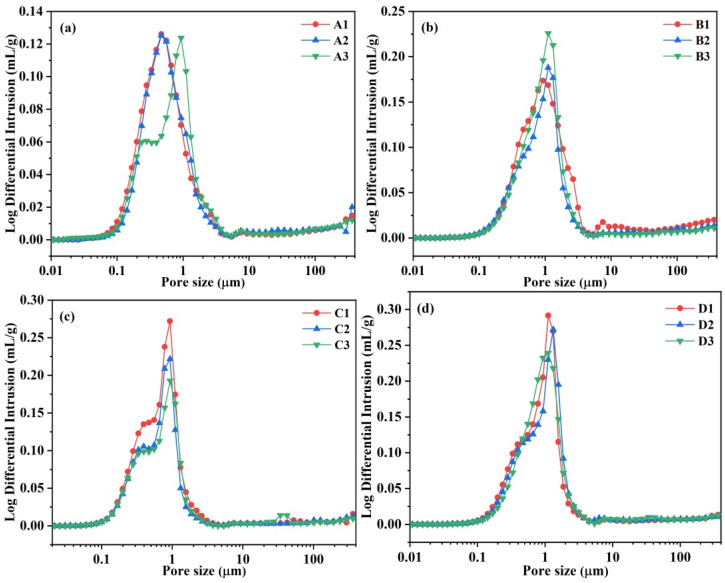
Pore size distribution of CA_6_-based insulating castables after firing at 1300 °C for 3 h: (**a**) A1–A3, (**b**) B1–B3, (**c**) C1–C3 and (**d**) D1–D3.

The pore size distribution of castables with different CAC contents measured through MIP is shown in Figure 8. A notable observation was the remarkable consistency in internal pore size distribution among samples within each group, with a pronounced peak in cumulative pore volume occurring within a range from 0.2 to 1 μm. Furthermore, according to Figure 8, the overall trend of pore size distribution reveals that internal pore characteristics varied with CAC content accordingly. Specifically, the peak of pore size distribution for sample A3 shifted to a greater size compared to other samples (Figure 8a). The reason of this phenomenon is that finer pores could be filled by cement with finer particle sizes. In castables prepared by adding an air-entraining agent, the distribution range of pore sizes for sample B2 was narrowest (Figure 8b). The peak of the pore size distribution of samples with alumina bubbles became lower with the increase in CAC content, as seen in Figure 8c. When both an air-entraining agent and alumina bubbles were added simultaneously, sample D2, also with 9 wt.% CAC content, displayed a relatively concentrated pore size distribution (Figure 8d).

**Figure 9:**
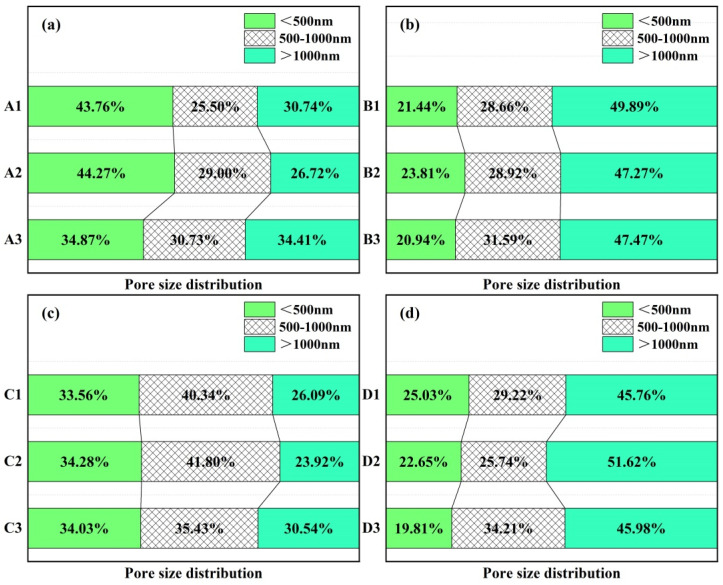
Statistical diagram of pore size distribution of CA_6_-based insulating castables: (**a**) A1–A3, (**b**) B1–B3, (**c**) C1–C3 and (**d**) D1–D3.

Figure 9 presents a statistical diagram depicting the percentage of pore volumes corresponding to pore sizes smaller than 500 nm, between 500 and 1000 nm, and larger than 1000 nm for each group. The pore size distribution exhibited significant variations among the sample groups. In samples with the absence of additives (A1–A3), the pore size predominantly fell below 500 nm. As the CAC content increased, the proportion of pores smaller than 500 nm initially rose but decreased significantly at 10 wt.% CAC, with a corresponding increase in pores larger than 1000 nm. The addition of an AEA (B1–B3) led to a substantial increase in pores larger than 1000 nm, accounting for nearly half of the total pore volume. In contrast, alumina-bubble-incorporating samples (C1–C3) exhibited a relatively balanced pore size distribution, with the proportion of pores between 500 and 1000 nm peaking at 9 wt.% CAC. Combining both additives (D1–D3) resulted in a pore size distribution skewed towards the 500–1000 nm range, with pores larger than 1000 nm constituting 45.8% to 51.6% of the total pore volume. Under the action of a combined air-entraining agent and alumina bubbles, the proportion of pore diameters larger than 1000 nm in sample D2 reached the maximum value of 51.62%, while the proportion of pores less than 500 nm was reduced below 20% if the cement content was 10 wt.%.

Following the fitting of the fractal dimensions for each sample, the calculated results are presented in Table 2. The pore fractal dimensions of the prepared castables exceeded 2.7, which was equivalent to the value of CAC-bonded castables [32]. In contrast, the castables prepared with the addition of an air-entraining agent and alumina bubbles exhibited lower fractal dimensions, indicating a relatively simple pore structure. As the CAC content increased, the fractal dimension of the castables prepared without additives decreased, and consequently, the complexity of their pore structure also decreased owing to the reduction in porosity derived from the filling effect of more cement. Specifically, at a CAC content of 10 wt.%, the fractal dimensions were lowest in sample A3. Similarly, at a CAC content of 8 wt.%, B1 and C1 exhibited the lowest fractal dimensions. Sample D2, with a CAC content of 9 wt.%, presented the lowest fractal dimensions. In general, the incorporation of alumina bubbles can make the pore structure more complex. Samples D1–D3, with lower fractal dimensions, had weaker strength, which was in good agreement with the results reported by Wang et al. [33].

### 3.7. Thermal Conductivity

**Figure 10:**
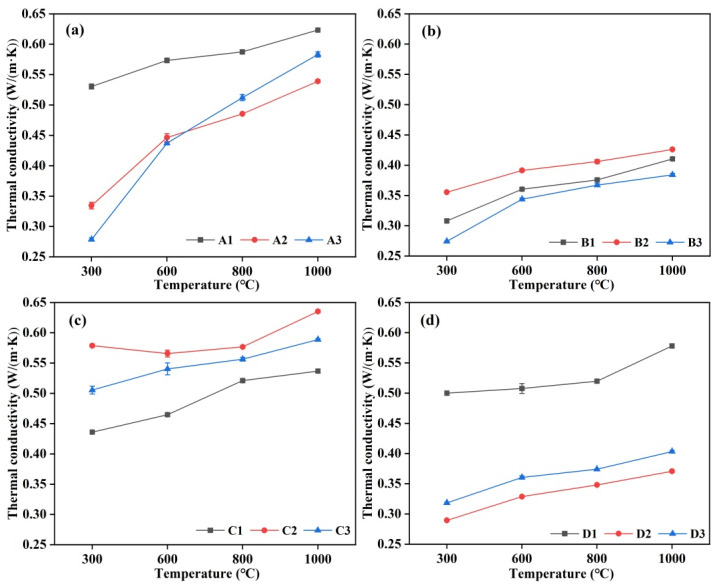
Thermal conductivity of CA_6_-based insulating castables after treated at 1300 °C for 3 h: (**a**) A1–A3, (**b**) B1–B3, (**c**) C1–C3 and (**d**) D1–D3.

The impact of varying cement content on the thermal conductivity of castables is illustrated in Figure 10. It can be seen that the thermal conductivity of the samples exhibited an upward trend as the test temperature increased, which is attributed to the enhanced thermal movement of atoms, coupled with increased heat conduction through the air within the pores and radiation between pore walls as the temperature rose. Furthermore, a comparative analysis of the thermal conductivity of each group revealed that an increase in CAC content significantly affected the thermal conductivity of the samples, although this effect was not confined to any particular group. The thermal conductivity data for samples at 1000 °C were used as a reference point, and the samples were subsequently arranged in ascending order based on their thermal conductivity values. Among the samples prepared without any additives, the order of thermal conductivity from lowest to highest was: A2 < A3 < A1. For the castables prepared with the addition of an air-entraining agent, the order of thermal conductivity from lowest to highest was: B3 < B1 < B2. The order of thermal conductivity of the castable samples prepared with alumina bubbles from lowest to highest was: C1 < C3 < C2. For the castable specimens prepared with the simultaneous addition of both an air-entraining agent and alumina bubbles, the order of thermal conductivity from lowest to highest was: D2 < D3 < D1. The samples exhibiting the lowest thermal conductivity in each group were A2, B3, C1, and D2, with corresponding CAC contents of 9 wt.%, 10 wt.%, 8 wt.% and 9 wt.%. Among them, the thermal conductivity of sample D2 at 1000 °C was as low as 0.37 W/(m·K).

It was notable that while the alteration of cement content affects the thermal conductivity of castables, the overall thermal conductivity of castables prepared with an AEA, as well as those prepared with an AEA and alumina bubbles, was lower. The low thermal conductivity of the samples was associated with porosities formed after the addition of AEA. Furthermore, the samples with low thermal conductivity at 1000 °C from castables prepared with different additives within each group were subjected to comparison and analysis. The samples were arranged in descending order of apparent porosity, with D2 exhibiting the lowest porosity, followed by B3, C1, and A2. It can be seen that the incorporation of AEA exerted a more pronounced influence on the thermal conductivity of the castables than the alteration of the cement content.

### 3.8. Grey Correlation Analysis

As a highly active branch within grey system theory, grey correlation analysis (GCA) enables effective qualitative comparisons of interaction relationships between various factors in a system [34]. The selection of the recognition coefficient directly influences computational results of correlation degrees, thereby affecting the credibility of system model evaluations and prediction outcomes. The detailed calculation formulas are as follows [35,36]:

The property parameter is selected as the original reference sequence:

X_0_ = {*x*_0_(1), *x*_0_(2),…, *x*_0_(m)} (2)

The influencing factor variables are listed as the original comparative sequences:

X*_i_*= {*x_i_*(1), *x_i_*(2), …, *x_i_*(m)} *i* = 1, 2, …, *n* (3)

Normalize the sequence as:

(4) $$x_{i}^{\prime }\left(k\right)=\frac{x_{i}\left(k\right)}{\bar{x_{i\left(k\right)}}}\;\;k=1,2,\ldots ,m\;\;i=1,2,\ldots ,n$$

The correlation coefficient *ξ_i_* of $x_{0}^{\prime }\left(k\right)$ and $x_{i}^{\prime }\left(k\right)$ can be calculated as:

(5) $$\xi_{i}\left(k\right)=\frac{\underset{i}{\min}\underset{k}{\min}\left|x_{0}^{\prime }\left(k\right)-x_{i}^{\prime }\left(k\right)\right|+\rho \;\underset{i}{\max}\underset{k}{\max}\left|x_{0}^{\prime }\left(k\right)-x_{i}^{\prime }\left(k\right)\right|}{\left\lceil x_{0}^{\prime }\left(k\right)-x_{i}^{\prime }\left(k\right)\right\rceil +\rho \;\underset{i}{\max}\underset{k}{\max}\left|x_{0}^{\prime }\left(k\right)-x_{i}^{\prime }\left(k\right)\right|}$$

where the recognition coefficient is *ρ*.

The grey correlation degree *φ_i_* of X_0_ and X*_i_* is calculated as:

(6) $$\varphi_{i}=\frac{1}{n}\sum_{i=1}^{n}\xi_{i}\left(k\right)$$

Following Lv Feng’s study [37], the recognition coefficients were determined as 0.35, 0.35, and 0.3, respectively. The grey correlation degrees of cement content, apparent porosity, pore fractal dimension, and pore size distribution fractions (across specific ranges) to CMOR, CCS, and thermal conductivity were calculated.

**Figure 11:**
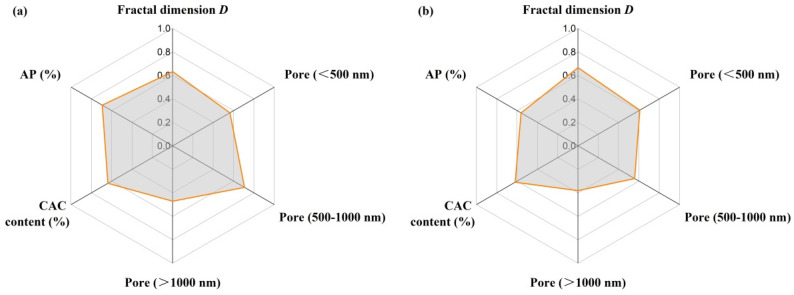
Grey correlation degree between other parameters and strength (CMOR (**a**) and CCS (**b**)) of castables after treatment at 1300 °C.

**Figure 12:**
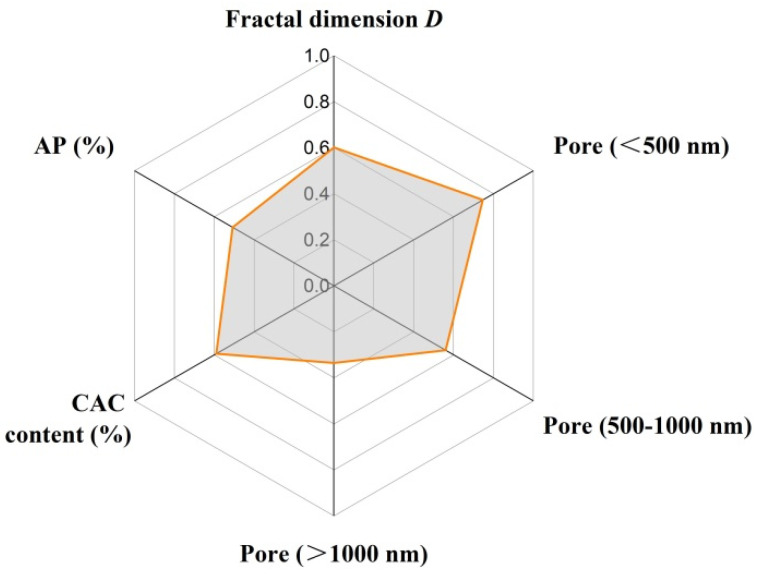
Grey correlation degree between other parameters and thermal conductivity of castables at 1000 °C.

Figure 11 presents the calculated grey correlation degrees between other parameters and strength based on GCA. The results demonstrated that the grey correlation degrees of pores with a size in the range of 500–1000 nm and apparent porosity were highest for CMOR, followed by CAC content and the pore fractal dimensions, while pore fractal dimensions had a maximal effect on CCS, and CAC content and pores smaller than 500 nm were the second and third related factors respectively. Grey correlation degrees between other parameters and the thermal conductivity of castables at 1000 °C are shown in Figure 12. The grey correlation degree of pores smaller than 500 nm was 0.75, suggesting that finer pores played an important role in thermal conductivity. Meanwhile, the grey correlation degrees of pore fractal dimensions and CAC content were around 0.6. Optimizing the performance of castables through adjusting the cement content can be achieved by changing the pore size distribution and complexity of pores.

## 4. Conclusions

The CAC content had a great influence on the performance of CA_6_-based insulating castables prepared by different pore-forming methods. The effects of CAC content (8–10 wt.%) on properties such as flowability, strength, and thermal conductivity were evaluated and systematically compared. The main conclusions were as follows:

(1) An increase in CAC content reduced the flowability of castables at 30 min and 60 min when adding alumina bubbles, but increased the initial flowability of castables prepared with AEA. The change in CAC content had no obvious effect on the phase composition of castables. After heat treatments at 1000 °C and 1300 °C, the primary phases in all samples were CA_6_ and α-Al_2_O_3_. Trace amounts of CA_2_ and CA persisted at 1000 °C, while only CA_2_ was present at 1300 °C.

(2) The increase in CAC content had a significant influence on the overall strength of samples. Especially for castables prepared by adding AEA, the strength improvement after heat treatment was more significant. The optimal strength was achieved when AEA and 9 wt.% CAC content were added, with the cold modulus of rupture (CMOR) and cold crushing strength (CCS) values at 17.5 MPa and 80.5 MPa, respectively. The factors with the highest grey correlation degree with CMOR and CCS were pores with a size in the range of 500–1000 nm and pore fractal dimensions, respectively.

(3) For castables prepared with alumina bubbles, increasing the CAC content did not reduce the thermal conductivity. However, for those containing air-entraining agents, higher cement content further decreased thermal conductivity. Additionally, the pore size distribution and pore fractal dimensions were influenced by variations in CAC content. Pores smaller than 500 nm had the highest correlation with the thermal conductivity of CA_6_-based insulating castables.

(4) Based on comprehensive analysis, it was suggested that an optimized CAC content of 9 wt.% can be determined for CA_6_-based insulating castables with the addition of AEA, which is applicable for prefabricated blocks used in stator posts. For the construction of walking beams and posts, it is better to choose the formulation containing alumina bubbles and 8 wt.% CAC. Low thermal conductivity and sufficient strength for the support lining of beams and posts in reheating furnaces are guarantees for energy savings in and longer service lives of refractories.

## Figures and Tables

**Table 1:** Formulations of samples (wt.%).

Raw Materials		Recipe Numbers											
		A1	A2	A3	B1	B2	B3	C1	C2	C3	D1	D2	D3
CA_6_	<5 mm	57	57	57	57	57	57	54	54	54	54	54	54
	<0.074 mm	20	19	18	20	19	18	20	19	18	20	19	18
Alumina bubbles (0.5–0.2 mm)		0	0	0	0	0	0	3	3	3	3	3	3
Calcined alumina (D_50_ = 5 μm)		8	8	8	8	8	8	8	8	8	8	8	8
Reactive alumina (D_50_ = 1.5 μm)		7	7	7	7	7	7	7	7	7	7	7	7
Calcium aluminate cement (Secar71)		8	9	10	8	9	10	8	9	10	8	9	10
Dispersant (WSM-M)		0.1	0.1	0.1	0.1	0.1	0.1	0.1	0.1	0.1	0.1	0.1	0.1
Air-entraining agent (AEA2066)		0	0	0	0.06	0.06	0.06	0	0	0	0.06	0.06	0.06
Foam stabilizer (MT 400 PFV)		0	0	0	0.02	0.02	0.02	0	0	0	0.02	0.02	0.02
Water addition		10.3	10.3	10.3	10.8	10.8	10.8	10.3	10.3	10.3	10.8	10.8	10.8

**Table 2:** Fractal dimensions *D* of pores in different samples.

Sample	A1	A2	A3	B1	B2	B3	C1	C2	C3	D1	D2	D3
Fractal dimensions	2.820	2.805	2.800	2.713	2.829	2.816	2.844	2.847	2.875	2.775	2.734	2.784

## Data Availability

The data are not publicly available.
